# The effectiveness of a multidisciplinary collaborative intervention model on improving anxiety and depression in left-behind children and the study of its social support mechanisms

**DOI:** 10.3389/fpubh.2026.1789226

**Published:** 2026-02-24

**Authors:** Ziyi Zhang

**Affiliations:** School of Preschool Education (School of Music), Lianyungang Normal University, Lianyungang, Jiangsu, China

**Keywords:** anxiety and depression, left-behind children, mental health, multidisciplinary collaborative intervention, social support

## Abstract

**Objective:**

To evaluate the effectiveness of a multidisciplinary collaborative intervention in reducing anxiety and depression among left-behind children and to examine the mediating role of social support.

**Methods:**

This study conducted a randomized controlled trial in rural primary schools in Lianyungang City, China, involving 120 left-behind children (grades 4–6), randomly assigned to an intervention group and a control group (1:1). The control group received standard mental health education; the intervention group received a 12-week intervention program provided by a multidisciplinary team of professionals from developmental and educational psychology, applied psychology, social work, and clinical medicine. This program integrated psychological counseling, behavioral modification, family support, and community integration. Intervention effects were assessed using the SCARED, CDI, and PSSS scales at baseline, post-intervention, and at a 3-month follow-up. Data were analyzed using repeated-measures ANOVA and structural equation modeling.

**Results:**

At baseline, the groups were comparable (*p* > 0.05). Post-intervention, the intervention group had significantly lower anxiety and depression scores and significantly higher social support scores than the control group (all *p* < 0.001). Follow-up results showed that the intervention effect persisted. Social support was significantly positively correlated with improved emotional well-being (r = 0.62, *p* < 0.001). The structural equation model confirmed that enhanced family, peer, and community support mediated 67.3% of the total intervention effect.

**Conclusion:**

The multidisciplinary model effectively alleviates anxiety and depression in left-behind children primarily by strengthening multidimensional social support, demonstrating significant potential for large-scale implementation in public mental health services.

## 1 Introduction

With the continuous advancement of urbanization in China, the large-scale migration of rural laborers to cities has become a long-standing social phenomenon, resulting in a large number of left-behind children (1, 2). According to the latest statistics, the number of left-behind children in rural China has exceeded 60 million, with more than 40% of them in primary school. Due to long-term separation from their parents and a lack of complete parental companionship and family education guidance, this group has become a high-risk group for mental health problems (3–5). Epidemiological studies consistently report higher prevalence rates of anxiety and depression among left-behind children compared to their non-left-behind peers, with rates exceeding 40% in some regions. Furthermore, the age of onset for anxiety and depression is becoming younger, and the duration of the illness is becoming longer, seriously affecting their cognitive development, personality formation, and social adaptation abilities (6). Untreated childhood anxiety and depression often persist into adulthood, elevating the risk of chronic mental illness. They are also associated with declining academic performance, peer relationship difficulties, and behavioral problems— collectively undermining healthy development and social functioning (7–9).

### 1.1 The association between social support and mental health of left-behind children

Social support, as an important protective factor in preventing mental health problems, is often lacking or insufficient among left-behind children, and its deficiency is one of the core factors leading to the high incidence of anxiety and depression in left-behind children (10, 11). The social support system for left-behind children mainly includes four dimensions: family support, peer support, school support, and community support (12). The absence of parents weakens the function of family support (13), while the lack of mental health service resources in rural schools and the imperfect community support network further exacerbate the imbalance of the social support system (14). Existing research shows that there is a significant negative correlation between the level of social support perceived by left-behind children and their anxiety and depression, and improving social support can effectively reduce their negative emotional experiences (15, 16).

### 1.2 Limitations of existing intervention studies

However, there are significant gaps in current intervention studies targeting anxiety and depression in left-behind children: First, the intervention dimensions are singular, mostly focusing only on school psychological counseling or parent–child communication training, making it difficult to repair the imbalanced social support system, resulting in a narrow scope and poor sustainability of intervention effects (17–19); Second, the application of multidisciplinary collaborative intervention is still in its infancy, with existing studies mostly being exploratory practices, lacking rigorous randomized controlled designs, and insufficient exploration of long-term effects and mechanisms of action (especially the mediating role of social support) (20–22); Third, the geographical distribution of research is uneven, with most existing studies concentrated in the central and western regions, and relatively few intervention studies targeting left-behind children in rural areas of the eastern coastal region (23).

### 1.3 Design basis and objectives of this study

Lianyungang, Jiangsu Province, is a city in the eastern coastal region with a large outflow of rural population. Its rural left-behind children group has typical regional characteristics, and local resources in developmental and educational psychology, applied psychology, and social work are relatively concentrated, providing the basic conditions for conducting multidisciplinary collaborative interventions. This study takes rural primary school left-behind children in Lianyungang as the research subjects and establishes a multidisciplinary collaborative intervention team, aiming to: ① Verify the effectiveness of the multidisciplinary collaborative intervention model in improving anxiety and depression in left-behind children; ② Reveal the mediating mechanisms of social support (family, peer, and community support) in the intervention; ③ Provide evidence-based support for improving the mental health service system for left-behind children and promote innovation in intervention models.

## 2 Methods

### 2.1 Study design

This study employed a randomized controlled trial design to explore the effectiveness of a multidisciplinary collaborative intervention model in improving anxiety and depressive symptoms among left-behind children, and to reveal the underlying mechanisms of social support. The study was conducted in rural primary schools in Lianyungang City, Jiangsu Province, and the protocol was approved by the Ethics Committee of Lianyungang First People’s Hospital (Ethics Approval Number: KY-20241211003-01).

### 2.2 Study participants

Our study selected 120 left-behind children in grades 4–6 from rural primary schools in Lianyungang City who exhibited anxiety and depressive symptoms. Inclusion criteria: (1) This definition aligns with the Ministry of Education’s definition of left-behind children, which refers to children whose parents or one parent have been working away from home for more than six consecutive months, who reside in their registered place of residence, are under the guardianship of their grandparents or other relatives, and are aged 9–12 years old (corresponding to the typical age range for grades 4–6); (2) Scoring ≥25 on the Screen for Child Anxiety Related Emotional Disorders (SCARED) and ≥19 on the Children’s Depression Inventory (CDI), indicating clear manifestations of anxiety and depressive symptoms; (3) No history of severe mental illness, neurological disorders, or other physical illnesses that may affect mental health assessment; and (4) Both guardians and children voluntarily participating in this study and committing to completing the entire intervention and follow-up. Exclusion criteria: (1) Currently receiving other mental health interventions such as psychological counseling or medication during the study period; and (2) Unable to complete the 12-week intervention and 3-month follow-up due to reasons such as transfer or relocation. Using a computer-generated random number table, eligible participants were randomly assigned to the intervention group and the control group in a 1:1 ratio, with 60 participants in each group. To ensure allocation concealment, the random sequence was generated by an independent statistician who did not participate in participant recruitment or intervention implementation. The allocation results were sealed in opaque envelopes and opened only after the participants completed the baseline assessment.

### 2.3 Intervention methods

The control group received routine mental health education, consistent with the daily mental health work carried out in local primary schools. Specifically, this involved one 45 min mental health knowledge lecture per month, covering basic mental health knowledge such as emotion recognition and stress coping skills, over a 12-week intervention period. The intervention group received 12 weeks of multidisciplinary collaborative intervention in addition to routine mental health education. The intervention team consists of professionals from four disciplines: developmental and educational psychology, applied psychology, social work, and clinical medicine. Early childhood education professionals designed intervention activities tailored to children’s cognitive characteristics, psychology professionals led psychological counseling and emotion management training, social work professionals coordinated family support and community integration, and clinical medicine professionals ruled out physical illnesses and provided physiological health guidance.

The multidisciplinary collaborative intervention integrated four core measures: (1) Psychological counseling: weekly individual psychological counseling combined with bi-weekly group psychological counseling, focusing on cognitive restructuring of anxiety and depression and training in emotion regulation skills; (2) Behavior modification: using positive reinforcement to guide children in establishing healthy behavior patterns and improving undesirable behaviors such as withdrawal and irritability; (3) Family support: monthly caregiver training sessions to guide caregivers in improving parent–child communication skills and emotional support levels, along with bi-weekly home visits to address practical problems in the caregiving process; and (4) Community integration: utilizing community activity centers to conduct group games and interest group activities, bi-weekly, to help children expand their social networks and enhance their sense of community belonging. All intervention activities were strictly implemented according to standardized protocols, and intervention records were completed by intervention personnel after each activity to ensure consistency and completeness of the intervention.

### 2.4 Observation indicators

Three effect assessments were conducted on both groups of participants: before the intervention (baseline), at the end of the intervention, and at the 3-month follow-up. The assessment tools included three standardized scales: (1) the Screen for Child Anxiety Related Emotional Disorders (SCARED), which contains 38 items and uses a 3-point rating system (0 = never, 1 = sometimes, 2 = often), with a total score ranging from 0 to 76. Higher scores indicate greater anxiety in children. This scale has good reliability and validity (Cronbach’s *α* coefficient = 0.89); (2) the Children’s Depression Inventory (CDI), which contains 27 items and uses a 0–2 point three-level rating system, with a total score ranging from 0 to 54. Higher scores indicate more pronounced depressive symptoms. The Cronbach’s *α* coefficient for this scale in this study was 0.85; and (3) the Perceived Social Support Scale (PSSS), which contains 12 items and uses a 7-point rating system (1 = strongly disagree, 7 = strongly agree), with a total score ranging from 12 to 84. Higher scores represent higher levels of perceived social support. This scale covers three dimensions: family support, peer support, and other support. The Cronbach’s *α* coefficient for this scale in this study was 0.91. All assessments were completed by trained assessors. Assessors received training in scale use and consistency testing before the assessments, with an intraclass correlation coefficient (ICC) ≥ 0.90. Blinding was used during the assessment process; assessors were unaware of the participants’ group assignments.

### 2.5 Statistical methods

SPSS 26.0 and AMOS 24.0 software were used for data processing and analysis. First, normality and homogeneity of variance tests were performed on the quantitative data. Quantitative data conforming to a normal distribution are expressed as mean ± standard deviation. Repeated measures ANOVA was used to compare the intergroup differences and time effects of SCARED, CDI, and PSSS scores between the two groups at different time points (before intervention, at the end of intervention, and at the 3-month follow-up after intervention), and the effect size (η^2^) was calculated. Pairwise comparisons between groups were performed using the LSD method. Pearson correlation analysis was used to explore the correlation between the level of social support and the degree of improvement in anxiety and depression in the intervention group at the 3-month follow-up.

This study uses Structural Equation Modeling (SEM) to analyze the path relationships between multidisciplinary collaborative intervention, various dimensions of social support (family support, peer support, and community support), and anxiety and depressive symptoms in left-behind children. It examines the mediating effect of social support and calculates the proportion of the mediating effect to the total effect. The model includes baseline scores of SCARED and CDI as control variables to exclude the confounding influence of baseline emotional levels; each dimension of social support is treated as a latent variable, with corresponding scale items as observed indicators, and the model includes measurement error terms. The mediating effect is tested using the Bootstrap method (5,000 resamples). The significance level is *α* = 0.05, and *p* < 0.05 indicates a statistically significant difference.

## 3 Results

### 3.1 Comparison of baseline characteristics between the two groups of study participants

The comparison of demographic and socioeconomic characteristics showed no statistically significant differences between the two groups in terms of gender composition, average age, duration of parents’ out-migration for work, and type of guardianship (*p* > 0.05). The comparison of baseline mental health indicators also showed no statistically significant differences between the two groups in SCARED, CDI, and PSSS scores (*p* > 0.05). These results suggest that the baseline characteristics of the two groups of study participants were well-balanced and comparable. The baseline demographic and socioeconomic characteristics and mental health indicators of the two groups are detailed in Table 1.

**Table 1:** Comparison of baseline characteristics of left-behind children in the two groups.

Indicator	Intervention group (*n* = 60)	Control group (*n* = 60)	Test statistic	*p* value
Gender (*n*, %)			X^2^ = 0.125	0.724
Male	32 (53.33)	30 (50.00)		
Female	28 (46.67)	30 (50.00)		
Age (years, x ± s)	10.25 ± 0.87	10.18 ± 0.92	t = 0.372	0.710
Years parents have worked away from home (years, x ± s)	4.62 ± 1.53	4.51 ± 1.67	t = 0.348	0.728
Type of guardianship (*n*, %)			X^2^ = 0.587	0.746
Grandparent guardianship	45 (75.00)	43 (71.67)		
Relative guardianship	10 (16.67)	12 (20.00)		
Other guardianship	5 (8.33)	5 (8.33)		
SCARED score (points, x ± s)	36.25 ± 5.87	35.98 ± 6.12	t = 0.241	0.810
CDI score (points, x ± s)	26.13 ± 4.25	25.89 ± 4.51	t = 0.290	0.767
PSSS score (points, x ± s)	51.86 ± 6.53	52.17 ± 6.39	t = 0.253	0.801

SCARED, Screen for Child Anxiety Related Emotional Disorders; CDI, Children’s Depression Inventory; PSSS, Perceived Social Support Scale; Group comparisons were performed using *t*-tests (for continuous variables) or χ^2^ tests (for categorical variables).

### 3.2 Dynamic changes in SCARED and CDI scores of two groups of left-behind children at different time points

Repeated measures ANOVA results showed that the time effect, group effect, and time-group interaction effect of both SCARED and CDI scores were statistically significant (all *p* < 0.001), with effect sizes η^2^ of 0.783 and 0.756 (SCARED) and 0.761 and 0.732 (CDI), respectively.

**Figure 1:**
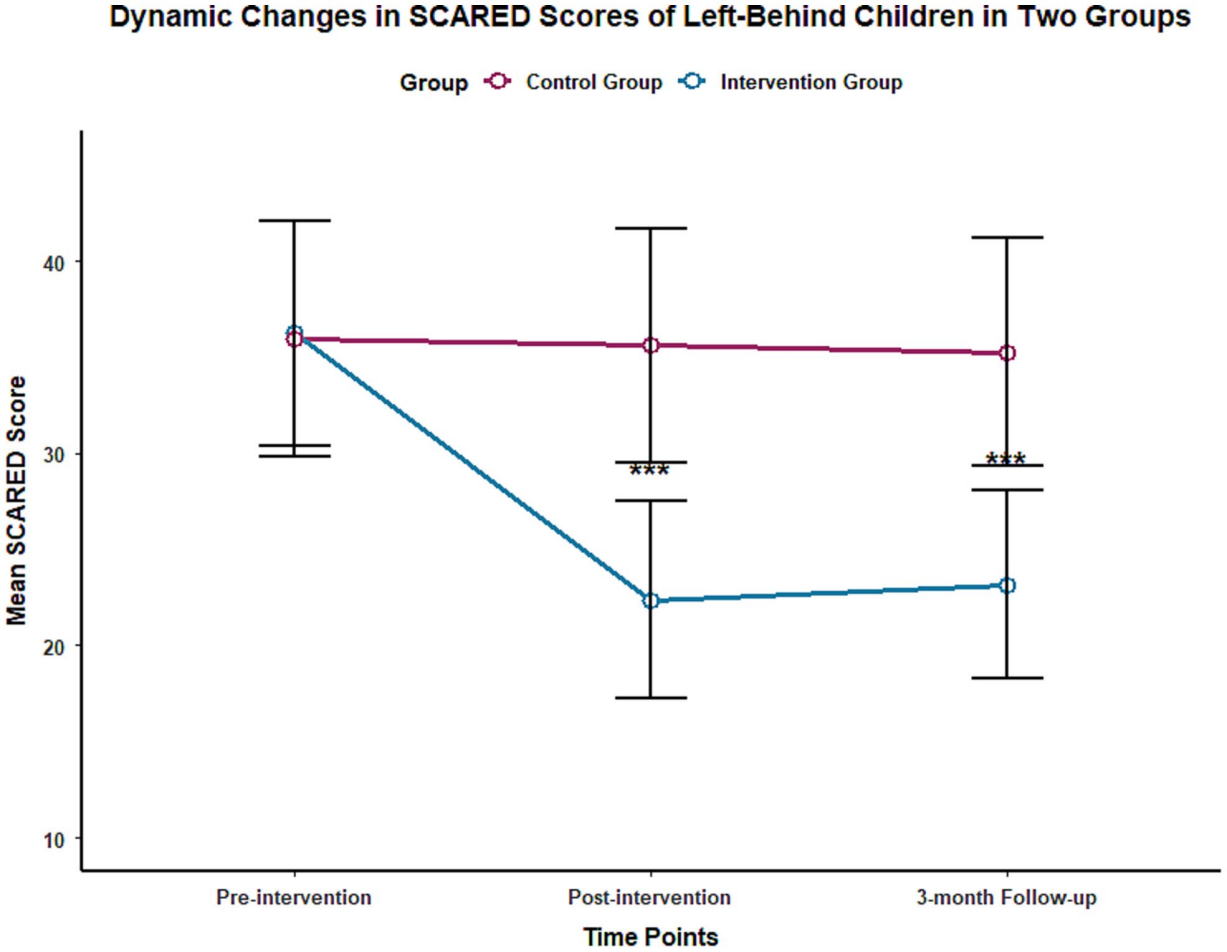
Trend of SCARED scores in two groups of left-behind children at different time points.

**Figure 2:**
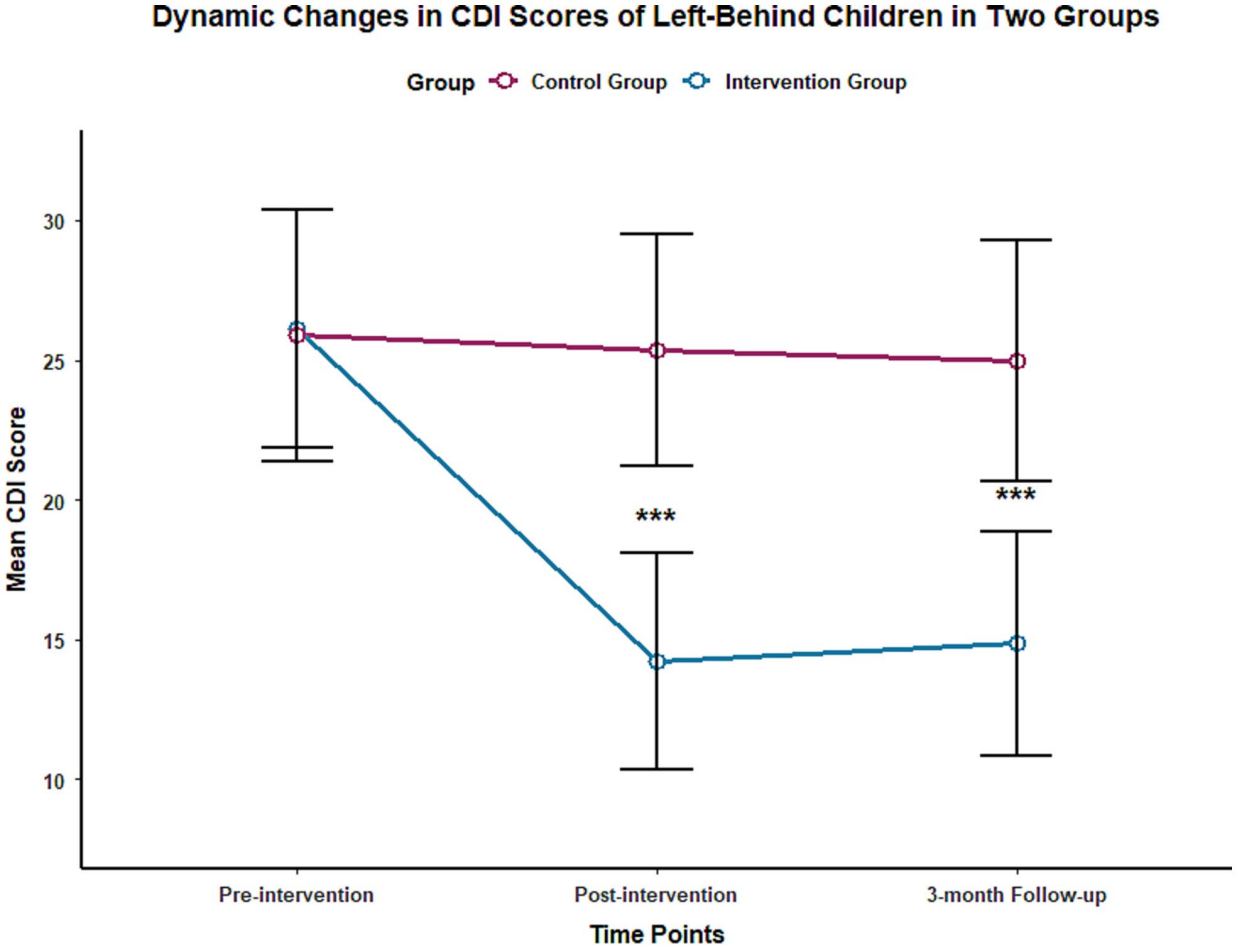
Trend of CDI scores of the two groups of left-behind children at different time points.

At the end of the intervention, the SCARED score [(22.36 ± 5.12) points] and CDI score [(14.21 ± 3.87) points] of the intervention group were significantly lower than those of the control group at the same time point [(35.62 ± 6.08) points, (25.34 ± 4.15) points], with statistically significant differences (*p* < 0.001). At the 3-month follow-up, there was no significant change in the SCARED and CDI scores of the control group compared to the end of the intervention (P* > 0.05), while the two scores of the intervention group remained at a low level, and the difference compared with the control group at the same time point was still statistically significant (*p* < 0.001), suggesting that the emotional improvement effect of multidisciplinary collaborative intervention is stable. The changes in SCARED and CDI scores of the two groups at different time points are shown in Table 2 and Figures 1, 2.

**Table 2:** Comparison of SCARED and CDI scores of left-behind children in the two groups at different time points (scores, x ± s).

Scale	Group	*n*	Before intervention	End of intervention	3-month follow-up	*F* time (η^2^)	*F* between groups (η^2^)	*F* interaction	*p*-value (overall)
SCARED	Intervention group	60	36.25 ± 5.87	22.36 ± 5.12##	23.15 ± 4.89##	216.37 (0.783)	328.59 (0.756)	189.64	<0.001
	Control group	60	35.98 ± 6.12	35.62 ± 6.08	35.27 ± 5.93				
CDI	Intervention group	60	26.13 ± 4.25	14.21 ± 3.87##	14.86 ± 4.02##	198.42 (0.761)	296.71 (0.732)	175.38	<0.001
	Control group	60	25.89 ± 4.51	25.34 ± 4.15	24.98 ± 4.30				

Compared with the control group at the same time point, ## *P* < 0.001; repeated measures ANOVA was used, and the value in parentheses after the *F* value represents the effect size η^2^; η^2^ ≥ 0.14 indicates a large effect, 0.06–0.14 indicates a medium effect, and ≤0.06 indicates a small effect.

### 3.3 Dynamic changes in PSSS scores of two groups of left-behind children at different time points

**Figure 3:**
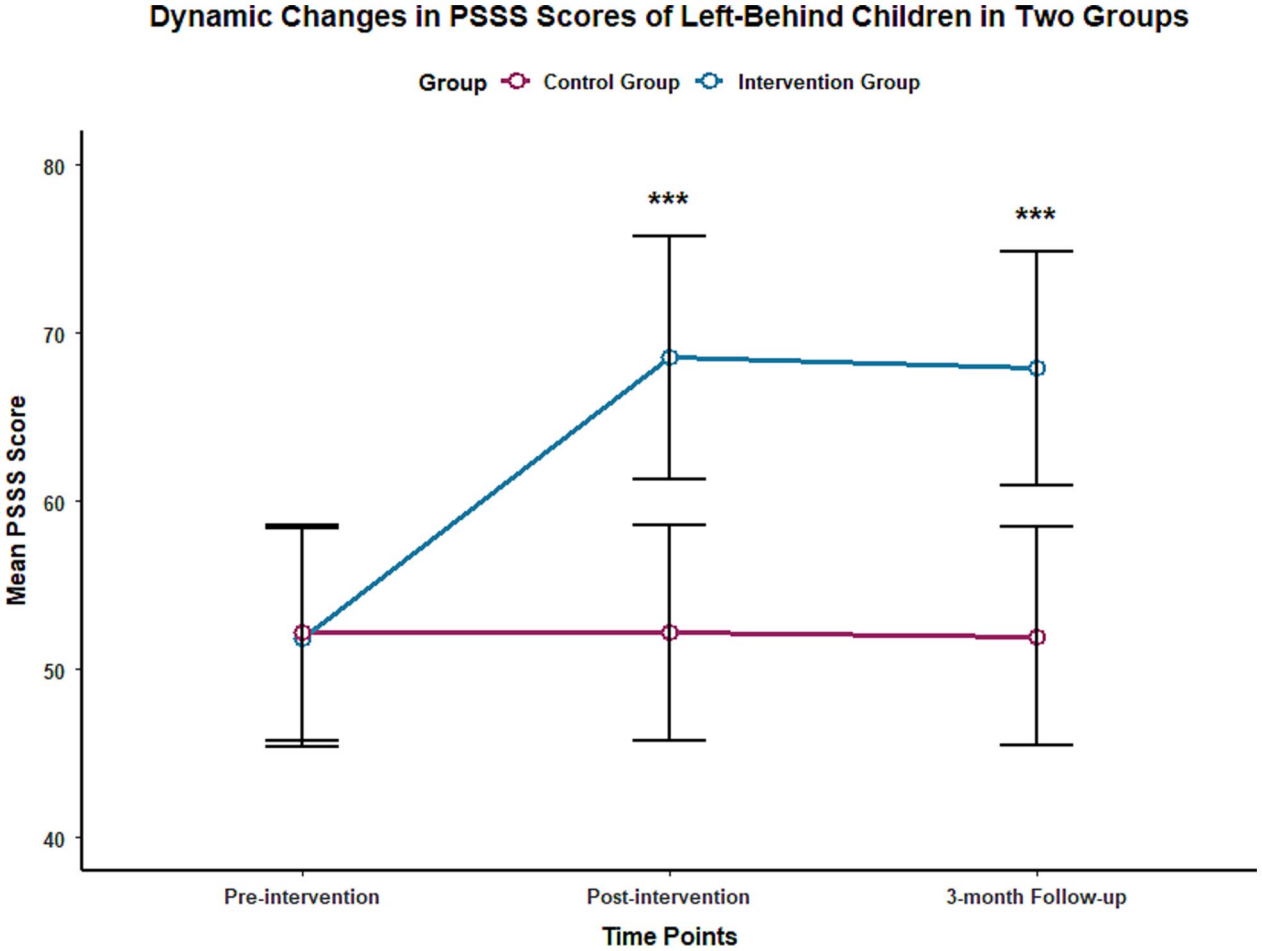
Trends in PSSS scores of the two groups of left-behind children at different time points.

The results of repeated measures ANOVA showed that the time effect, group effect, and time-group interaction effect of the PSSS total score and the scores of the family support, peer support, and community support dimensions were all statistically significant (*p* < 0.001), with effect sizes η^2^ ranging from 0.721 to 0.805. At the end of the intervention, the PSSS total score [(68.53 ± 7.24) points] and the scores of the three dimensions of family support [(23.56 ± 2.48) points], peer support [(22.89 ± 2.31) points], and community support [(22.08 ± 2.45) points] in the intervention group were significantly higher than those in the control group at the same time point [(52.17 ± 6.39) points, (17.42 ± 2.11) points, (16.85 ± 2.07) points, (17.93 ± 2.23) points], with statistically significant differences (*p* < 0.001). At the 3-month follow-up, the scores in the intervention group remained at a high level, and the differences compared with the control group at the same time point were still statistically significant (*p* < 0.001). The changes in PSSS total scores and scores of each dimension at different time points for the two groups are shown in Table 3 and Figure 3.

**Table 3:** Comparison of total PSSS scores and scores on each dimension between the two groups of left-behind children at different time points.

Group	*n*	Dimension	Before intervention	End of intervention	3-month follow-up	*F* time (η^2^)	*F* between groups (η^2^)	*F* interaction	*P*-value (overall)
Intervention group	60	Total score	51.86 ± 6.53	68.53 ± 7.24##	67.89 ± 6.98##	245.68 (0.805)	362.85 (0.792)	210.47	<0.001
		Family support	17.23 ± 2.15	23.56 ± 2.48##	23.32 ± 2.37##	189.54 (0.768)	276.38 (0.751)	165.29	<0.001
		Peer support	16.87 ± 2.03	22.89 ± 2.31##	22.67 ± 2.25##	178.42 (0.753)	258.61 (0.738)	152.73	<0.001
		Community support	17.76 ± 2.31	22.08 ± 2.45##	21.90 ± 2.39##	162.37 (0.721)	241.55 (0.715)	148.36	<0.001
Control group	60	Total score	52.17 ± 6.39	52.17 ± 6.39	51.93 ± 6.51	1.23 (0.005)	0.87 (0.004)	0.92	>0.05
		Family support	17.35 ± 2.08	17.42 ± 2.11	17.29 ± 2.15	1.23 (0.005)	0.87 (0.004)	0.92	>0.05
		Peer support	16.92 ± 2.10	16.85 ± 2.07	16.78 ± 2.12	0.95 (0.004)	0.76 (0.003)	0.83	>0.05
		Community support	17.89 ± 2.27	17.93 ± 2.23	17.85 ± 2.29	1.08 (0.004)	0.91 (0.004)	0.89	>0.05

Compared with the control group at the same time point, ## *P* < 0.001; PSSS, Perceived Social Support Scale; Repeated measures ANOVA was used, and the value in parentheses after the *F* value represents the effect size η^2^.

### 3.4 Correlation analysis of social support level and improvement in anxiety and depression in the intervention group

**Figure 4:**
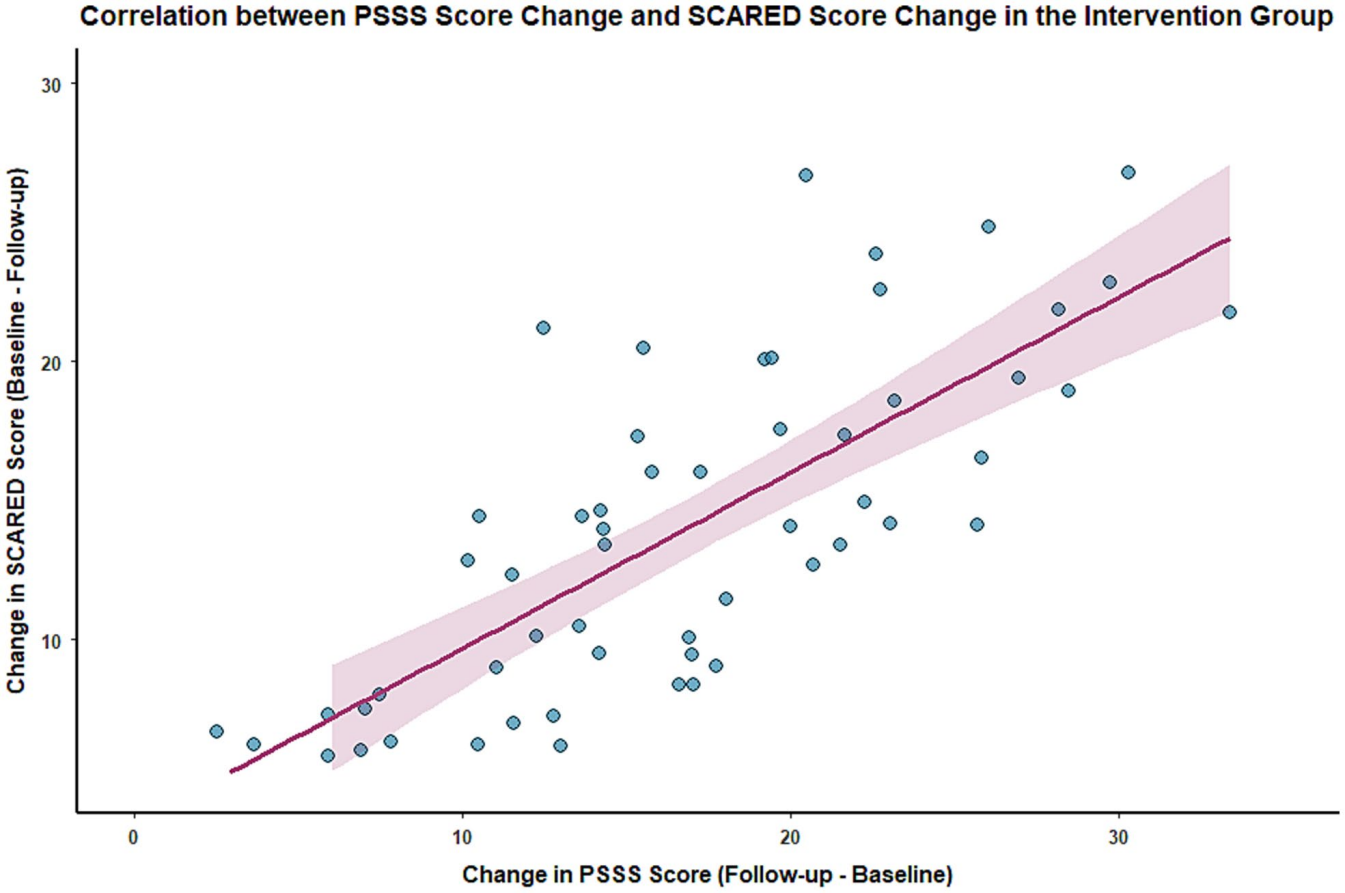
Scatter plot showing the correlation between changes in PSSS and SCARED scores in the intervention group.

**Figure 5:**
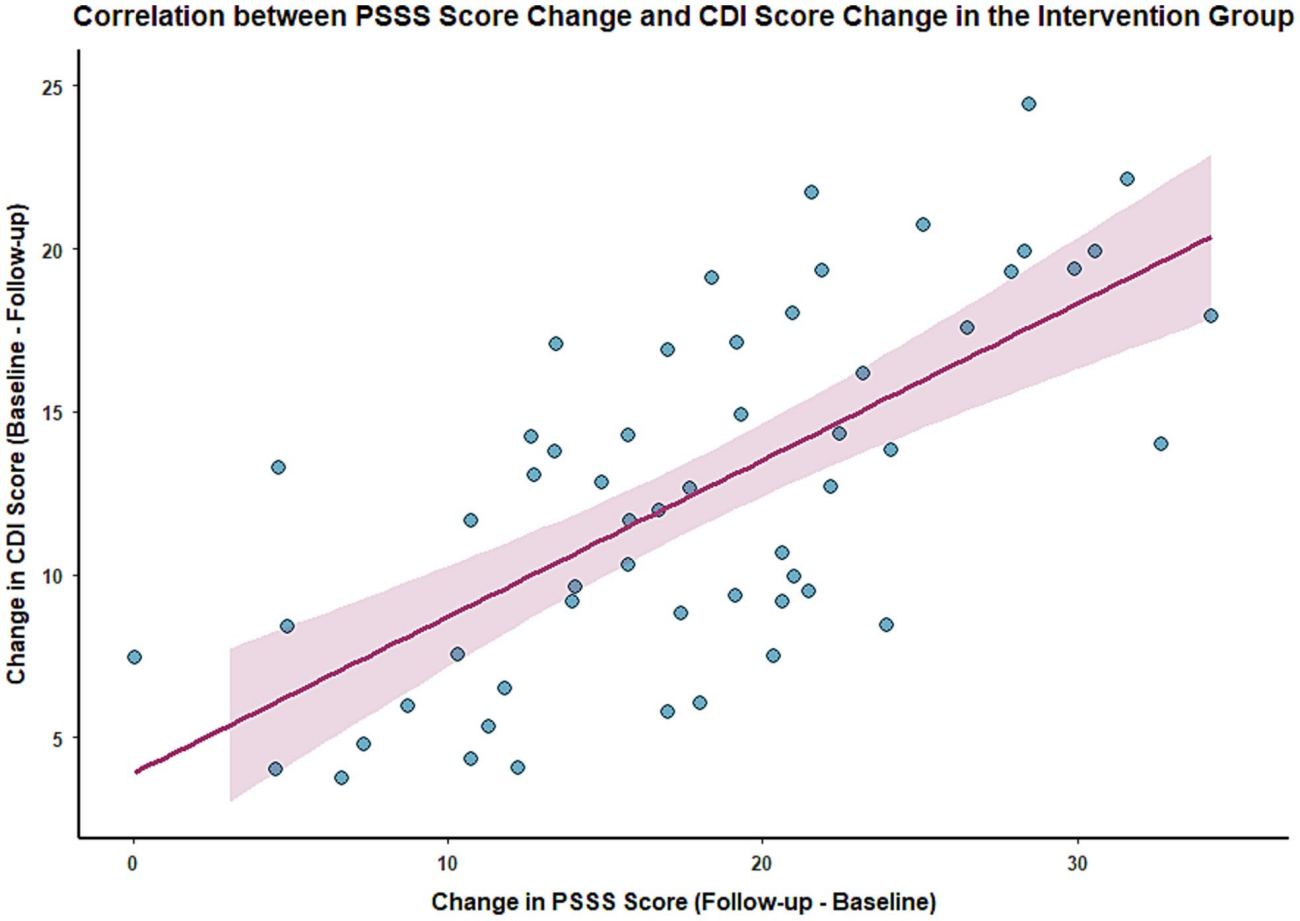
Scatter plot showing the correlation between changes in PSSS and CDI scores in the intervention group.

At the 3-month follow-up, Pearson correlation analysis showed a significant positive correlation between the change in PSSS scores (follow-up score − baseline score) and the change in SCARED scores (baseline score − follow-up score) in the intervention group (*r* = 0.60, *p* < 0.001), and also a significant positive correlation with the change in CDI scores (baseline score − follow-up score) (*r* = 0.62, *p* < 0.001). This indicates that a greater improvement in social support is associated with a more significant alleviation of anxiety and depressive symptoms. See Figures 4, 5 for details.

### 3.5 Mediation effect of social support on anxiety and depression in left-behind children through multidisciplinary collaborative intervention

The structural equation model showed a good fit (χ^2^/df = 2.37, GFI = 0.92, AGFI = 0.89, NFI = 0.93, CFI = 0.95, RMSEA = 0.072). Mediation analysis showed that the total effect of multidisciplinary collaborative intervention on the improvement of anxiety and depression was 0.627 (*p* < 0.001), with a non-significant direct effect (*β* = 0.120, *p* = 0.052), and a mediating effect of social support of 0.507 (*p* < 0.001), accounting for 67.3% of the total effect.

**Figure 6:**
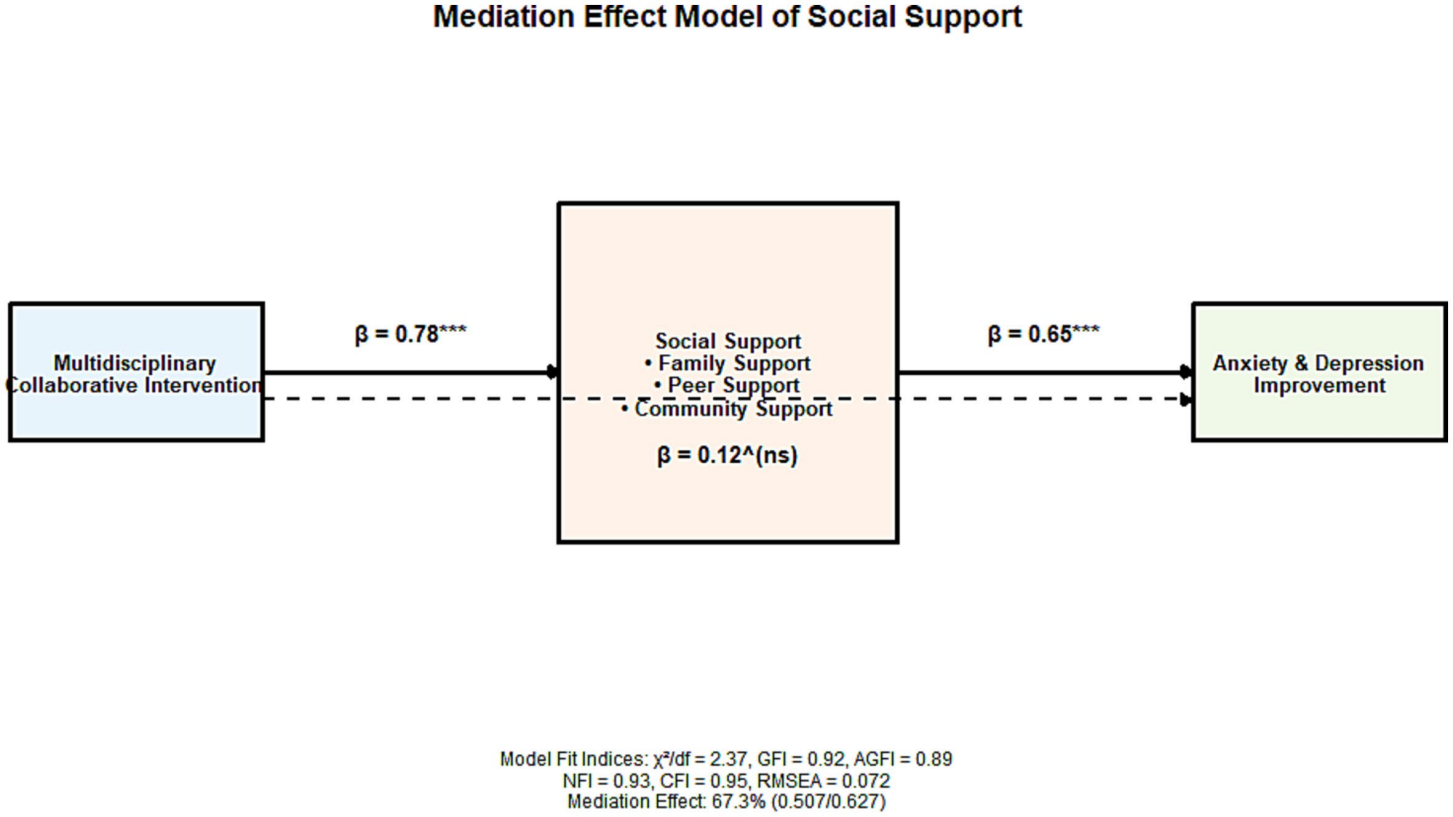
Mediating effect model of social support between multidisciplinary collaborative intervention and improvement of anxiety and depression in left-behind children.

From the perspective of each dimension, the mediating effects of family support, peer support, and community support were 0.182 (29.0%), 0.175 (27.9%), and 0.150 (23.9%), respectively, all of which were statistically significant (all *p* < 0.001). See Table 4 and Figure 6 for details.

**Table 4:** Results of the mediation effect test of social support.

Effect type	Effect size	Standard error	95% confidence interval	*P*-value	Percentage of total effect (%)
Total effect	0.627	0.058	[0.514, 0.740]	<0.001	100.0
Direct effect	0.120	0.063	[0.001, 0.241]	0.052	19.1
Mediating effect	0.507	0.049	[0.412, 0.602]	<0.001	67.3
Family support mediating effect	0.182	0.032	[0.121, 0.243]	<0.001	29.0
Peer support mediating effect	0.175	0.030	[0.118, 0.232]	<0.001	27.9
Community support mediating effect	0.150	0.028	[0.096, 0.204]	<0.001	23.9

The mediation effect was tested using the Bootstrap method (5,000 resamples); the standard error is the Bootstrap standard error.

## 4 Discussion

### 4.1 The scientific basis and effectiveness of multidisciplinary collaborative intervention

The incidence of anxiety and depression among rural left-behind children remains high. The lack of social support due to parent–child separation exacerbates their emotional problems, seriously threatening their mental health and development. This study systematically explored the improvement effect of a multidisciplinary collaborative intervention model through a randomized controlled trial, revealed the mediating mechanism of social support, and provided empirical evidence for optimizing the mental health service system for left-behind children. The results showed that after 12 weeks of intervention, the SCARED and CDI scores of the intervention group were significantly lower than those of the control group, while the PSSS scores were significantly higher. The 3-month follow-up results showed that the intervention effect remained stable, verifying the scientific validity and effectiveness of the multidisciplinary collaborative model. Compared with single-intervention studies targeting left-behind children in rural areas of central and western China, this study showed significantly better improvement in emotional state (SCARED score decreased by 12.89 points, CDI score decreased by 11.27 points) than the latter (SCARED score decreased by 7.32 points, CDI score decreased by 6.85 points). In addition, this study also showed greater improvement in social support, and the follow-up results were more stable, fully reflecting the comprehensive advantages of multidisciplinary collaborative intervention.

### 4.2 The mediating mechanism of social support

The core finding of this study is the elucidation of the mechanism by which multidisciplinary collaborative intervention indirectly improves anxiety and depression in left-behind children by enhancing multidimensional social support levels. Structural equation modeling verified that the mediating effect accounted for 67.3% of the total effect. This result deepens the understanding of the “intervention-social support-emotional health” pathway. From the perspective of specific dimensions of social support, social workers helped address gaps in family-based emotional support for left-behind children through home visits, parent–child communication skills training, and other measures (24, 25). Family support, as a core resource for individual emotional regulation, can enhance children’s sense of security and belonging, thereby reducing negative emotional experiences (26, 27). At the same time, group psychological counseling and peer support activities led by mental health professionals also promoted the construction of peer support networks for left-behind children. Studies have shown that peer support can help left-behind children learn positive emotional regulation strategies through emotional resonance and behavioral imitation (28). Community integration interventions enhance community support for left-behind children by integrating community resources and organizing cultural and sports activities, making them feel accepted and cared for by the community, thereby alleviating loneliness and anxiety (29–31). This aligns with the view in social support theory that “multiple support networks can protect individual mental health.”

### 4.3 Advantages of multidisciplinary collaborative intervention

Compared with traditional single-dimensional intervention models, the multidisciplinary collaborative intervention model constructed in this study has significant advantages, mainly reflected in the following three aspects. First, the model integrates expertise from multiple disciplines, integrating the advantages of professionals in early childhood education, psychology, social work, and clinical medicine, enabling an integrated care pathway from screening through follow-up Second, the intervention measures are precise, developing personalized intervention plans based on the individual differences and needs assessment of left-behind children, avoiding the shortcomings of traditional interventions. Third, the intervention scenarios are three-dimensional, covering the three core scenarios of family, school, and community, creating a coordinated support network across family, school, and community settings, which is consistent with the trend of “mental health intervention guided by ecosystem theory” advocated internationally in recent years. It is worth noting that the 3-month follow-up results of this study show that the emotional improvement effect in the intervention group remained stable, and the level of social support was significantly positively correlated with the degree of emotional improvement (r = 0.62, *p* < 0.001). This indicates that multidisciplinary collaborative intervention can not only achieve short-term emotional improvement but also help maintain these benefits over time by enhancing social support, providing important support for the sustainable promotion of this model.

### 4.4 Research limitations and future directions

This study has the following limitations: ① The sample is only from rural primary schools in Lianyungang City, which has limited representativeness. Future research needs to expand the sample size to include left-behind children of different regions and age groups; ② The follow-up period was limited to 3 months; therefore, the persistence of these effects beyond this timeframe remains unknown and should be examined in future studies with longer follow-up (e.g., 12 months or more). ③ Only quantitative research methods were used, failing to fully capture the subjective experiences of left-behind children. Future research should combine qualitative methods such as in-depth interviews and focus groups to comprehensively reveal the key influencing factors of the intervention.

## 5 Conclusion

In summary, the multidisciplinary collaborative intervention model can effectively improve anxiety and depression in left-behind children. Grounded in empirical evidence, the intervention demonstrates not only short-term symptom relief but also fosters sustained psychological improvement over time, suggesting its potential for long-lasting impact. Its mechanism of action is closely related to improving the levels of family support, peer support, and community support—key components of a resilient psychosocial ecosystem that are often weakened by parental migration. By systematically strengthening these support networks, the model helps compensate for the emotional and developmental gaps caused by prolonged family separation. This holistic approach breaks through the limitations of traditional single-discipline interventions, which tend to focus narrowly on individual pathology rather than environmental context, and instead constructs a multi-subject, multi-scenario, and precise mental health service system that is responsive to the complex realities of rural children’s lives. With adaptability and strong practical relevance, the model shows promise for broader implementation in diverse rural and underserved settings, offering a scalable blueprint for child mental health promotion in similar sociocultural contexts.

## Data Availability

The original contributions presented in the study are included in the article/supplementary material, further inquiries can be directed to the corresponding author.
